# Identifying Network Biomarkers in Early Diagnosis of Hepatocellular Carcinoma via miRNA–Gene Interaction Network Analysis

**DOI:** 10.3390/cimb45090466

**Published:** 2023-09-10

**Authors:** Zhiyuan Yang, Yuanyuan Qi, Yijing Wang, Xiangyun Chen, Yuerong Wang, Xiaoli Zhang

**Affiliations:** 1 School of Artificial Intelligence, Hangzhou Dianzi University, Hangzhou 310018, Chinaxlzhang@alumni.hust.edu.cn (X.Z.); 2 School of Physics and Key Laboratory of Molecular Biophysics of the Ministry of Education, Huazhong University of Science and Technology, Wuhan 430074, China

**Keywords:** hepatocellular carcinoma, network biomarker, microRNA–gene interaction network

## Abstract

Background: Hepatocellular carcinoma (HCC) is a highly heterogeneous cancer at the histological level. Despite the emergence of new biological technology, advanced-stage HCC remains largely incurable. The prediction of a cancer biomarker is a key problem for targeted therapy in the disease. Methods: We performed a miRNA–gene integrated analysis to identify differentially expressed miRNAs (DEMs) and genes (DEGs) of HCC. The DEM–DEG interaction network was constructed and analyzed. Gene ontology enrichment and survival analyses were also performed in this study. Results: By the analysis of healthy and tumor samples, we found that 94 DEGs and 25 DEMs were significantly differentially expressed in different datasets. Gene ontology enrichment analysis showed that these 94 DEGs were significantly enriched in the term “Liver” with a statistical *p*-value of 1.71 × 10^−26^. Function enrichment analysis indicated that these genes were significantly overrepresented in the term “monocarboxylic acid metabolic process” with a *p*-value = 2.94 × 10^−18^. Two sets (fourteen genes and five miRNAs) were screened by a miRNA–gene integrated analysis of their interaction network. The statistical analysis of these molecules showed that five genes (CLEC4G, GLS2, H2AFZ, STMN1, TUBA1B) and two miRNAs (hsa-miR-326 and has-miR-331-5p) have significant effects on the survival prognosis of patients. Conclusion: We believe that our study could provide critical clinical biomarkers for the targeted therapy of HCC.

## 1. Introduction

Hepatocellular carcinoma (HCC) is a high-mortality disease with no effective curable treatments and is the major cause of cancer death worldwide [1]. This disease is also known as the fifth most common tumor worldwide. HCC is a primary malignancy of the liver and occurs predominantly in patients with underlying chronic liver disease. Some cases of HCC are associated with viruses, such as hepatitis B virus and hepatitis C virus [2]. Surgery and liver transplantation are usually thought to be potentially curative treatments for patients with early hepatocellular carcinoma. Currently, high-throughput sequencing technology has been developed to identify gene expression profiling and distinct genetic alterations in HCC patients [3]. The progression of this disease appears to be closely related to gene mutations and oncogenic pathways. The increased understanding of the heterogeneous molecular pathogenesis of HCC has led to significant developments in novel targeted therapies.

Biomarkers are essential for optimized clinical decision-making by providing prognostic information and predicting response to specific therapies. Understanding the prognostic biomarkers is central to quality oncology care. With biomarkers, the abnormal biological processes and disease states of each patient can be accurately predicted. Alpha-fetoprotein (AFP) is one of the commonly used markers for early diagnosis of HCC [4]. AFP is a protein that the liver makes when its cells are growing and dividing to make new cells. Recent advancements in genomics have ushered in a new era of precision medicine [5]. Novel attempts have led to the development of a growing list of corresponding biomarkers in different types of cancers, such as lung cancer [6], cholangiocarcinoma [7] and brain tumor glioma [8]. Therefore, identifying specific biomarkers in early stage has great clinical significance in the treatment of HCC.

In the field of molecular biology, miRNA in regulating cancer pathogenesis has been a major achievement over the past decade [9]. This type of RNA comprises nearly 5% of transcriptome and regulates approximately 25% of genes in humans [10]. The miRNA can inversely modulate gene expression via directly inducing messenger RNA degradation by base pairing with complementary sites in the 3′-untranslated regions. Scientific evidence indicates that specific miRNAs are associated with the pathological features of HCC, such as hsa-miR-766 and hsa-miR-203. The hsa-miR-766 promotes cancer progression of HCC cells by targeting NR3C2 in vivo and has been reported to be an effective biomarker for liver cancer [11]. The hsa-miR-203 serves as a tumor suppressor in many types of cancer including esophageal cancers, breast cancer and hepatocellular carcinoma. This miRNA was reported to play an important role in the progression and carcinogenesis of HCC by targeting with the MAPK signaling pathway [12]. However, most of these studies only focus on the miRNA expression of the samples but do not take the miRNA–gene interaction into consideration; thus, a miRNA–gene integrated analysis was applied to identify novel biomarkers in this study. 

Here, we carried out a large, comprehensive multiomic study for the identification of miRNAs associated with genes in HCC. The expression profiles of miRNAs were elucidated from several independent cohorts of HCC cases and noncancer controls. Furthermore, we report a miRNA–gene integrated model based on the multiomic expression level of HCC and identify several useful molecules in this study.

## 2. Materials and Methods

### 2.1. Screening Differentially Expressed Genes and miRNAs

To find suitable datasets for the analysis, we searched the keywords “liver cancer” in the Gene Expression Omnibus (GEO) database [13] in April 2022. By filtering the results with “Homo sapiens” in organism and “Expression profiling by array” in study type, we obtained 871 datasets. We then checked these 871 datasets one by one according to the following criteria: (a) the dataset was published in the past two years; (b) the dataset must include enough effective samples (total number of samples ≥ 10, normal samples ≥ 5 and HCC samples ≥ 5; (c) the dataset must include a sufficient number of sequenced miRNAs (≥500) or genes (≥5000).

Based on the above-mentioned criteria, three datasets (GSE101685, GSE176271, GSE164760) were obtained for gene expression analysis. In order to analyze the differences between normal and HCC samples, we used GEO2R tool to analyze the three datasets (GSE101685, GSE176271, GSE164760) and obtained the differentially expressed genes (DEGs). The criterion for DEGs was set as a statistical *p*-value ≤ 0.05. We then compared the difference in the DEGs among these three datasets and drew a Venn diagram to show our results. 

**Figure 1:**
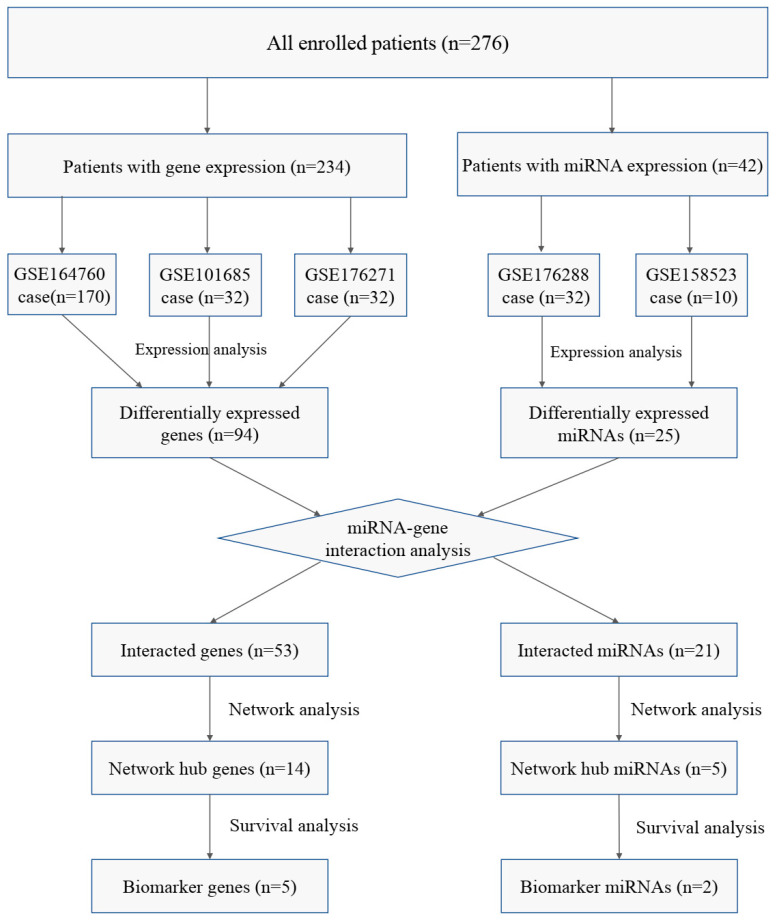
Patient flow diagram of this study.

Similarly, two datasets containing miRNA expression (GSE176288, GSE158523) were obtained for miRNA expression analysis. GSE176288 contains 32 available samples, while GSE158523 contains 10 available samples. The criterion for differentially expressed miRNAs (DEMs) was set as a statistical *p*-value ≤ 0.05. The common DEM section of GSE176288 and GSE158523 was then screened. The patient flowchart is shown in Figure 1.

### 2.2. Functional Enrichment Analysis 

Functional enrichment analysis could provide significant biological insights into the characteristics of studied genes. Metascape is a web-based tool providing a comprehensive gene annotation and analysis resource for scientists [14]. DAVID provides a comprehensive set of tools to identify enriched gene ontology terms and biological processes of studied genes [15]. By Metascape and DAVID analyses, we obtained the function enrichment results of DEGs.

### 2.3. miRNA–Gene Integrated Analysis

The miRWalk is a common tool to predict miRNA–target interaction by an effective machine learning algorithm [16]. For target prediction, the lower binding value indicated the higher binding possibility of the miRNA and gene. We defined the binding values above −15 kcal/mol as high energy, the binding values below −25 kcal/mol as low energy and the binding values between −25 and −15 kcal/mol as medium energy. This method can effectively classify the target prediction level of miRNA. To enhance our prediction results, the miRTarbase [17] database was also applied to predict the target genes of miRNAs. By using miRWalk and miRTarbase, the connection between DEGs and DEMs was built. The metabolic pathways of miRNA–target genes were analyzed using the WikiPathways database [18] and miRPathDB tool [19].

### 2.4. Construct miRNA–Gene Integrated Network

To strengthen the completeness of the network, the DEG–DEG interaction network was also obtained from STRING database [20]. The two interaction networks (DEG–DEG and DEM–DEG) were then combined to construct a full miRNA–gene network. The Cytoscape software is one of the most common network biology analysis tools [21]. This tool was applied to analyze the network topology and node attributes. The interaction network was considered as a graph composed of the edges of miRNA–gene and gene–gene. The edge betweenness centrality is defined as the number of the shortest paths that go through an edge in a graph [22]. The edge betweenness value is a commonly used index to represent the centrality of edges and is often used in the analysis of biological networks [23]. Based on this value, some hub miRNAs and genes of the network were screened for further analysis.

### 2.5. Survival Analysis of Hub Nodes

Survival analysis is a branch of statistics to study the expected duration and is widely used in cancer research. Survival analysis is especially helpful in analyzing the association of a biomarker and disease. The Cancer Genome Atlas (TCGA) database provides publicly available clinical and high-throughput genomic data for various types of cancers. GEPIA server was utilized to analyze our selected genes in TCGA database [24]. KMplot was applied to conduct the survival analysis of these genes [25] with statistics cutoff *p*-value ≤ 0.05. The gene satisfying this criterion was then retained for further discussion.

## 3. Results

### 3.1. Screening Differentially Expressed Genes

**Figure 2:**
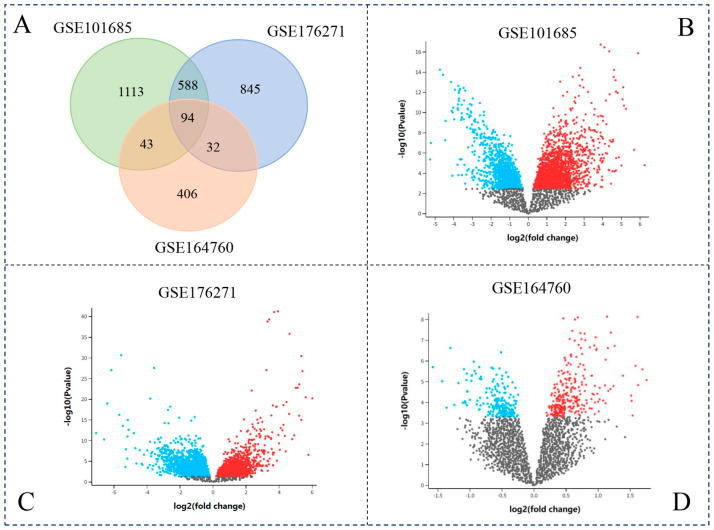
Analysis of differentially expressed genes in HCC. (**A**) Venn diagram analysis of three datasets; (**B**) volcano plot of GSE101685; (**C**) volcano plot of GSE176271; (**D**) volcano plot of GSE164760.

We screened the DEGs in each dataset using as the criterion *p*-value ≤ 0.05 by the GEO2R tool. GEO2R is a tool that performs differential expression testing on most microarray datasets in NCBI. A set of 1838 DEGs was identified in the dataset GSE101685, while a group of 1559 DEGs was obtained in the dataset GSE176271. In addition, 575 genes were differentially expressed in the dataset GSE164760, which is relatively fewer than in the other two datasets. A set of 94 DEGs was present in all three datasets (Figure 2A) and denoted as core DEGs (cDEGs). Most of these cDEGs showed an extremely low *p*-value (≤0.001) when compared to the genes in normal and cancer patients (Table S1). 

### 3.2. Function Enrichment 

We performed a function enrichment analysis to investigate these 94 cDEGs by the Metascape and DAVID databases. By a tissue-specific analysis of these DEGs, we found that most of these genes could be enriched in different types of tissues. The largest proportion (57.45%) of DEGs was significantly enriched in the keyword “Liver”, with a significant *p*-value = 1.71 × 10^−26^ (Table 1), which indicated that our identified DEGs are mostly liver-specific genes. The term “monocarboxylic acid metabolic process” (GO:0032787) was significantly overrepresented in our selected DEGs with a *p*-value = 1 × 10^−18^ in biological process, which indicated that some of these genes were involved in energy metabolism. The monocarboxylic acid metabolic process is a type of nutrient homeostasis process; thus, these DEGs may reflect the rapid conversion of the liver function in association with the response to a variety of external environments [26].

Besides, the term “fatty acid metabolic process” (GO:0006631) was significantly enriched in our DEGs with a *p*-value = 2.82 × 10^−11^, which suggested that these genes may contribute to the growth of cancer cells. It is well known that the liver is the central organ for fatty acid metabolism. Fatty acids accrue in liver by hepatocellular uptake from the plasma and by de novo biosynthesis. Fatty acids are eliminated by oxidation within the cell or by secretion into the plasma within low-density lipoproteins [27]. These enrichment results indicated that our identified DEGs were extremely related to liver.

### 3.3. Identification of cDEM

**Figure 3:**
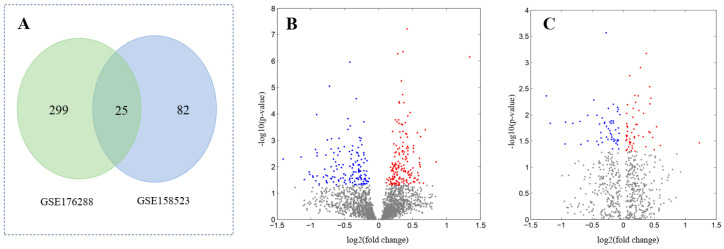
Analysis of differentially expressed miRNAs in hepatocellular carcinoma. (**A**) Venn diagram analysis of two datasets; (**B**) volcano plot of GSE176288; (**C**) volcano plot of GSE158523.

Two HCC datasets (GSE176288, GSE158523) were found in the NCBI database containing miRNA expression in normal and HCC samples. The results showed that 324 miRNAs in GSE176288 and 107 miRNAs in GSE158523 were significantly differentially expressed. Using the Venn diagram, 25 differentially expressed miRNAs (DEMs) were found shared in these two datasets and denoted as core DEMs (cDEMs) (Figure 3A). Because these miRNA samples were taken from different tissues, this resulted in relatively inconsistent data between them. In addition, currently, there are approximately 2000 known miRNAs in human. The number is significantly lower than the gene number (about 23,000) in human. Therefore, the number of differentially expressed miRNAs was much lower than the number of differentially expressed genes (Table S2).

### 3.4. cDEG–cDEM Interaction Network

Based on the miRWalk, we built an interaction between the cDEGs and cDEMs. A set of 20 miRNAs (cDEMs) was found to interact with cDEGs. These 20 miRNAs were categorized into 19 different families. Only miR-106 family was observed to have more than one member (hsa-miR-106a-5p, hsa-miR-106b-5p) (Table 2). Most of the interactions were predicted with very low binding energy, for example, hsa-miR-2277-5p interacted with CYP39A1 by a critical low binding value −29.3 kcal/mol. CYP39A1 belongs to the member of the cytochrome P450 family, which catalyzes many reactions involved in drug metabolism. This gene is a liver-specific gene with a female-preferential expression and strongly suppressed HCC development [28]. Our findings suggest that these genes could serve as novel targets for the biological therapy for and diagnosis of HCC.

### 3.5. Target Enrichment Analysis of cDEM

The targeted genes of miRNAs (cDEMs) were enriched by the WikiPathways database and miRPathDB tool. The targets of nine miRNAs (hsa-miR-451a, hsa-miR-106a-5p, hsa-miR-106b-5p, hsa-miR-17-5p, hsa-miR-15b-5p, has-miR-18a-5p, has-miR-376c-3p, has-miR-326, hsa-miR-381-3p) were found to be enriched in the pathway of “Hepatitis C and Hepatocellular Carcinoma” (Table 3). A total of 30 genes were identified as putative targets of hsa-miR-381-3p with a *p*-value = 0.049. In addition, five genes were identified as hsa-miR-451a targets, with an extremely low *p*-value = 2.3 × 10^−6^. In a previous study, hsa-miR-451a was reported to inhibit the proliferation and migration of hepatocellular carcinoma cells by targeting gene YWHAZ [29]. The YWHAZ protein plays an important role in tumor progression and is involved in many signal transduction pathways in liver cancer [30]. We suggest that other miRNAs in this table may also affect the proliferation of HCC, which needs further investigation in the future.

### 3.6. Network Analysis of the cDEG–cDEM Network

**Figure 4:**
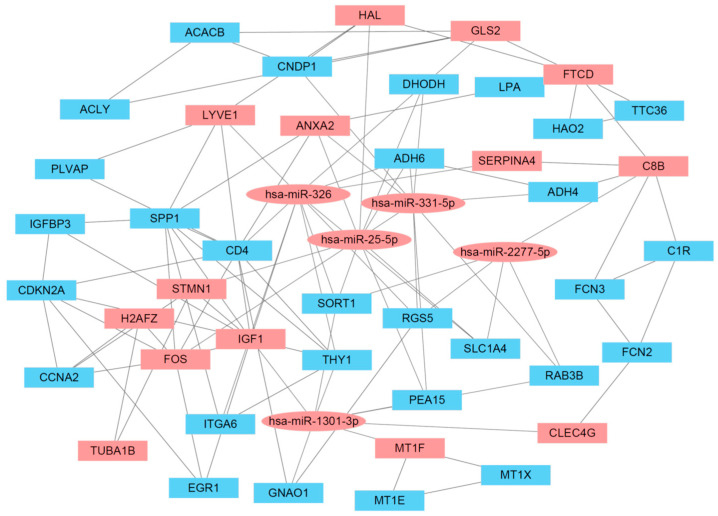
The interaction network of cDEMs and cDEGs in HCC. The red blocks are the identified nodes with high betweenness scores.

The cDEG–cDEM network was built and visualized by Cytoscape. Previous studies indicated that betweenness centrality is a robust attribute in the identification of target genes in the molecule–molecule network [31]. The betweenness centrality score is calculated based on the shortest path between a node and other nodes in the network. Based on the betweenness centrality score of the cDEG–cDEM network, we screened out fourteen key genes (ANXA2, C8B, CLEC4G, FOS, FTCD, GLS2, H2AFZ, HAL, IGF1, LYVE1, MT1F, STERPINA4, STMN1, TUBA1B) and five key miRNAs (hsa-miR-1301-3p, hsa-miR-25-5p, hsa-miR-2277-5p, hsa-miR-326, hsa-miR-331-5p) (Table 4). These genes and miRNAs are highlighted with red blocks in Figure 4. The edge of FOS/H2AFZ interaction showed a betweenness score of 512.84, while the edge of hsa-miR-1301-3p/MT1F interaction showed a betweenness score of 387.96. The edge with high betweenness score indicated the most influential molecules in the network involved in spreading the disease.

### 3.7. Validation of Hub Genes by GEPIA

**Figure 5:**
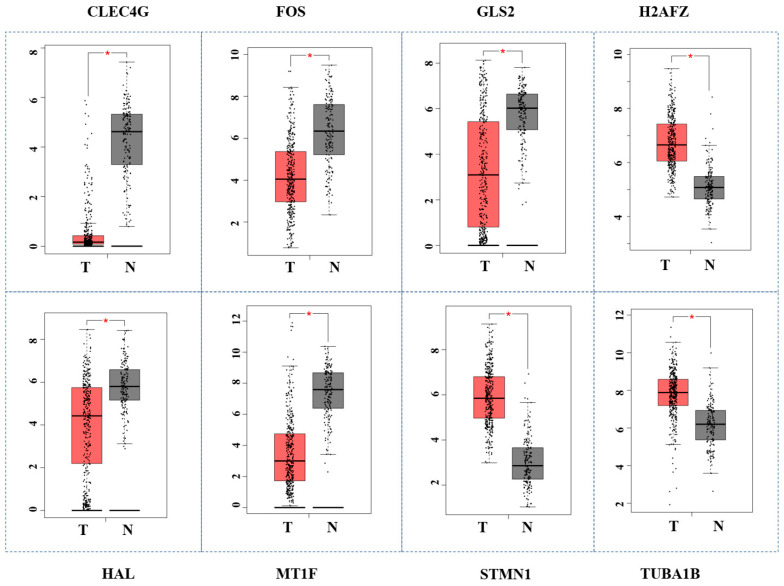
The validation of hub genes by GEPIA. The star indicates the significant difference between normal and HCC samples.

**Figure 6:**
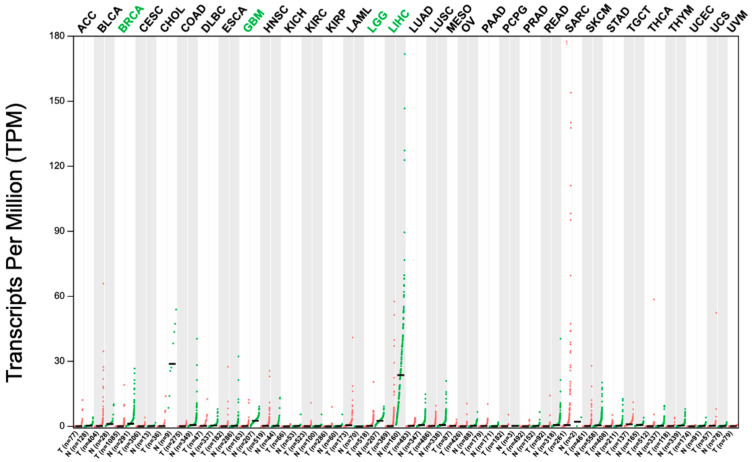
Dot plot of CLEC4G gene in different types of cancer. The red dots indicate the gene expression in tumor tissue, while the green dots indicate the gene expression in normal tissue.

In the above paragraph, we screened out 14 key genes with high betweenness scores in the miRNA–gene interaction network. To verify the reliability of these fourteen genes, we applied the GEPIA tool to test their performance in the TCGA database. The results indicated that eight genes (CLEC4G, FOS, GLS2, H2AFZ, HAL, MT1F, STMN1, TUBA1B) showed significant differential expression between normal and HCC samples in the TCGA database with a *p*-value ≤ 0.05 (Figure 5). Four genes (CLEC4G, MT1F, STMN1, H2AFZ) obviously diverged between HCC and normal tissue. A dot plot of the expression levels of these genes in all cancer types was drawn. The results showed that the expression levels of CLEC4G in liver cancer were much higher than the expression levels in other cancer types (Figure 6). The CLEC4G gene encodes a glycan-binding receptor, a member of the C-type lectin family, which plays an important role in the immune response. C-type lectin proteins are pattern recognition receptors located on the immune cells that are active in the cell signaling pathways. CLEC4 is associated with the infiltration of various immune cells and is crucial for the development of HCC. The low expression level of CLEC4G may signify the low activity of immune cells in HCC patients. This result indicated that CLEC4G could serve as a specific biomarker for the prediction of HCC progression.

### 3.8. Survival Analysis of Hub Genes Using KMplot

**Figure 7:**
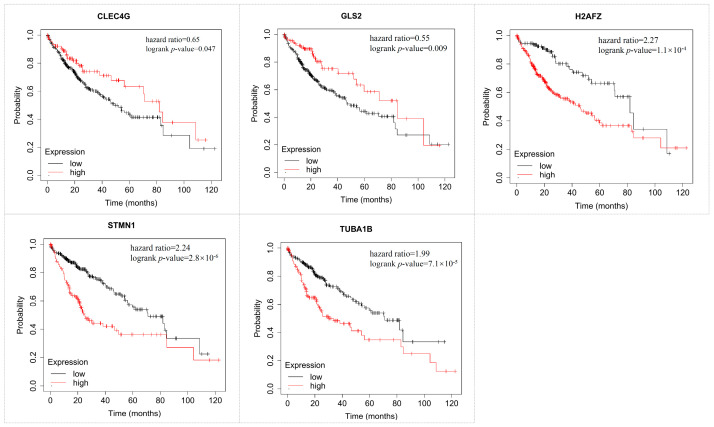
Survival analysis of hub genes by the KMplot tool. The red line indicates high expression, and the black line indicates low expression.

**Figure 8:**
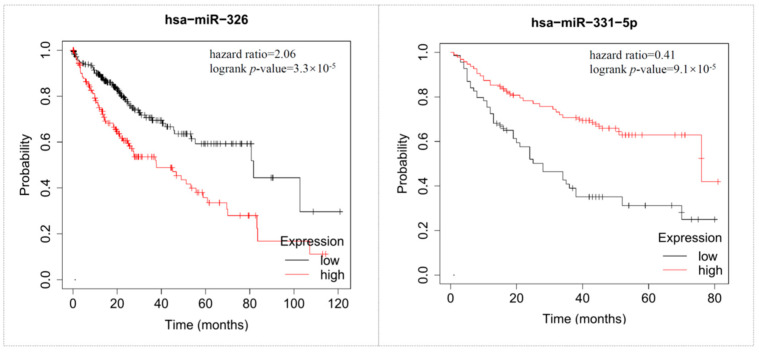
Survival analysis of hub miRNAs by the KMplot tool. The red line indicates high expression, and the black line indicates low expression.

Survival analysis could provide reliable scientific results in clinical outcomes with a high level of confidence, and this process was conducted by the KMplot. We found that nine genes (ANXA2, CLEC4G, FTCD, GLS2, H2AFZ, IGF1, STERPINA4, STMN1, TUBA1B) showed a significant impact on the poor survival prognosis of HCC patients with a *p*-value ≤ 0.05. Among them, five genes (CLEC4G, GLS2, H2AFZ, STMN1, TUBA1B) had specific expression in HCC and a significant effect on patient survival (Figure 7). These five genes could be considered critical molecules in the development of HCC. In addition, two miRNAs (has-miR-326 and has-miR-331-5p) have a significant impact on the survival prognosis of this cancer (Figure 8). We believe these miRNAs can also be used as reliable biomarkers in the early detection of hepatocellular carcinoma.

## 4. Discussion

Hepatocellular carcinoma (HCC) is a leading cause of cancer-related deaths. Because of difficulties in early diagnosis and lack of targeted drugs, the survival rate of HCC is extremely low. Due to the genetic diversity and personal discrepancies, the existence of therapy has greatly limited the progress in early detection and molecular classification of HCC. Based on the progression of molecular biology, a bioinformatic analysis brings a new dimension to targeted treatment and the possibility of pre-clinical screening of tumors. Alternative biomarkers to treat this disease are urgently needed.

MicroRNAs have been regarded as potential epigenetic mechanisms partaking in the pathogenesis of HCC. The dysregulation of miRNAs has been related to poor outcomes in patients with this type of cancer [32]. In recent years, novel approaches to cancer treatment have been based on microRNAs, small noncoding RNA molecules that play a crucial role in cancer progression by regulating gene expression. It has been reported that the dysregulation of miRNA was found in the progression of HCC, such as hsa-miR-155 and hsa-miR-27a-3p. The high expression level of hsa-miR-155 has been related to the microvascular invasion of HCC patients. The expression of hsa-miR-155 has been elevated in tumor tissues from HCC patients with liver transplantation [33]. The hsa-miR-27a-3p overexpressed in mesenchymal stem cells could suppress golgi membrane proteins to inhibit the progression of this disease [34]. 

However, previous works on identifying biomarkers do not take the miRNA–gene interaction network into consideration. Network biology offers a powerful means to identify more robust biomarkers. The network-based approach exploits observations that genes with similar phenotypic roles tend to co-localize in a specific region of a protein–protein interaction network. Based on this feature, many network biomarkers could be predicted. For example, Kong et al. applied a machine learning approach to predict network biomarkers related to immune checkpoint inhibitors in patients [35]. Therefore, constructing a network to analyze biomarkers becomes an effective method for studying complex diseases.

In this study, we established a miRNA–gene interaction network for biomarker identification in HCC. Five datasets (GSE176288, GSE158523, GSE101685, GSE176271, GSE164760) were obtained from the NCBI database. Two groups (25 cDEMs and 94 cDEGs) were identified between HCC and adjacent normal tissues in the corresponding datasets, respectively. The network of cDEM–cDEG was constructed based on their interaction predicted by miRWalk2.0. By the topological analysis of the miRNA–gene interaction network, five miRNAs (hsa-miR-1301-3p, hsa-miR-25-5p, hsa-miR-2277-5p, hsa-miR-326, hsa-miR-331-5p) and fourteen genes (ANXA2, C8B, CLEC4G, FOS, FTCD, GLS2, H2AFZ, HAL, IGF1, LYVE1, MT1F, STERPINA4, STMN1, TUBA1B) were found with the highest betweenness scores in the interaction network, meaning that they are located in the center of the network. These genes and miRNAs could possibly serve as critical biological molecules in the cell differentiation and migration of the disease. Through the gene expression analysis by the GEPIA and survival analysis by the KMplot, two miRNAs (hsa-miR-326 and has-miR-331-5p) and five genes (CLEC4G, GLS2, H2AFZ, STMN1, TUBA1B) were further picked out. These seven molecules could be used as novel biomarkers for early diagnosis in HCC patients. The following are some examples of detailed descriptions.

The hsa-miR-326 has been previously reported to participate in chemotherapy resistance and embryonic development in many cancer types. The low expression of has-miR-326 is dramatically related to unfavorable prognosis and metastasis in HCC. A similar phenomenon was also discovered for hsa-miR-331-5p. Researchers found that the downregulation of hsa-miR-331-5p can lead to anticancer drug resistance to doxorubicin by targeting *p*-glycoprotein [36]. We may predict the prognosis of HCC patients by detecting the expression level of these miRNA biomarkers and artificially inhibiting cancer growth by their mimics.

The CLEC4G gene, which belongs to C-type lectin receptors, showed regulatory roles on immune cell to affect cell activity. Recent studies have indicated that the expression of CLEC4G affects the development and microenvironment of tumors [37]. GLS2 exhibits a tumor-suppressive function by inhibiting Rac1 activity, which in turn inhibits the migration, invasion and metastasis of malignancy cells. A previous study showed that GLS2 is a key glutaminolysis synthase implicated in activities consistent with HCC suppression [38]. H2AFZ, which belongs to the H2A family, regulated DNA replication and cell cycle signaling by several cancer-related transcription factors in this disease [39]. H2AFZ may restrain liver cancer cell proliferation and cause many alternative splicing events. This study has demonstrated that STMN1 upregulation promotes the occurrence and development of liver cancer via activating a signaling pathway [37]. It is also reported that overexpressing STMN1 was closely associated with vascular invasion, drug resistance and shorter survival time in cancer patients in vivo [40]. TUBA1B, a type of tubulin protein, was reported to associate with the formation of the cytoskeleton and immune cell infiltration [41]. Thus, based on the above analysis, we have high confidence that our identified miRNAs and genes can be used as novel biomarkers for the understanding and diagnosis of HCC.

## 5. Conclusions

We applied a robust pipeline of the miRNA–gene interaction network analysis to investigate the molecule expression in HCC samples. Through the topological analysis of the miRNA–gene interaction network, we screened five genes (CLEC4G, GLS2, H2AFZ, STMN1, TUBA1B) and two miRNAs (hsa-miR-326 and has-miR-331-5p) according to their network betweenness scores. These molecules could be used as novel biomarkers for the diagnosis and survival prognosis of hepatocellular carcinoma patients.

## Figures and Tables

**Table 1:** Function enrichment analysis of 94 cDEGs by Metascape and DAVID.

Category	Term	Count	Proportion	*p*-Value
UP_TISSUE	Liver	54	57.45%	1.71 × 10^−26^
UP_TISSUE	Kidney	15	15.96%	1.77 × 10^−02^
UP_TISSUE	Plasma	13	13.83%	1.88 × 10^−08^
UP_TISSUE	PCR rescued clones	9	9.57%	3.28 × 10^−02^
UP_TISSUE	Hippocampus	7	7.45%	2.52 × 10^−02^
UP_TISSUE	Fetal liver	6	6.38%	3.46 × 10^−03^
UP_TISSUE	Myometrium	2	2.13%	4.25 × 10^−02^
GO Biological Processes	Monocarboxylic acid metabolic process	21	22.34%	2.94 × 10^−18^
GO Biological Processes	Alcohol metabolic process	13	13.83%	1.99 × 10^−11^
GO Biological Processes	Fatty-acid metabolic process	13	13.83%	2.82 × 10^−11^
Reactome Gene Sets	Biological oxidation	12	12.77%	5.20 × 10^−12^
GO Biological Processes	Olefinic compound metabolic process	11	11.70%	9.69 × 10^−13^
GO Biological Processes	Terpenoid metabolic process	10	10.64%	5.26 × 10^−13^
GO Biological Processes	Isoprenoid metabolic process	10	10.64%	2.98 × 10^−12^
GO Biological Processes	Cellular hormone metabolic process	10	10.64%	7.48 × 10^−12^
GO Biological Processes	Epoxygenase P450 pathway	6	6.38%	2.03 × 10^−11^
Reactome Gene Sets	Synthesis of hydroxyeicosatetraenoic acids	5	5.32%	3.27 × 10^−11^

**Table 2:** DEG–DEM interaction prediction. This table only shows one interaction of one DEM. The full table is shown in Table S3.

No.	miRNA	Transcribed mRNA	Gene Symbol	Binding Energy
1	hsa-miR-106a-5p	NM_001361	DHODH	−22.8
2	hsa-miR-106b-5p	NM_001102470	ADH6	−21.4
3	hsa-miR-1180-3p	NM_001145454	STMN1	−27.5
4	hsa-miR-1301-3p	NM_033304	ADRA1A	−26.9
5	hsa-miR-136-5p	NM_001237	CCNA2	−20.8
6	hsa-miR-15b-5p	NM_001280797	GLS2	−19.0
7	hsa-miR-18a-5p	NM_152545	RASGEF1B	−20.9
8	hsa-miR-2277-5p	NM_016593	CYP39A1	−29.3
9	hsa-miR-25-5p	NM_018281	ECHDC2	−24.2
10	hsa-miR-324-5p	NM_001257	CDH13	−23.9
11	hsa-miR-326	NM_001289033	SERPINA4	−29.0
12	hsa-miR-331-5p	NM_001306171	ADH4	−27.4
13	hsa-miR-374b-3p	NM_001144911	CLEC4M	−17.1
14	hsa-miR-379-5p	NM_001363587	CYP4A11	−22.4
15	hsa-miR-17-5p	NM_001297576	PEA15	−18.7
16	hsa-miR-301a-3p	NM_001297576	PEA15	−16.4
17	hsa-miR-369-5p	NM_001205228	SORT1	−16.8
18	hsa-miR-376c-3p	NM_152545	RASGEF1B	−18.0
19	hsa-miR-381-3p	NM_001311160	THY1	−23.3
20	hsa-miR-451a	NM_001311160	THY1	−17.0

**Table 3:** Enrichment analysis of targeted cDEM gene by WikiPathways. The full table is shown in Table S4.

miRNA	Database	Pathway	Hits	*p*-Value
hsa-miR-451a	WikiPathways	Hepatitis C and Hepatocellular Carcinoma	5	2.3 × 10^−6^
hsa-miR-106a-5p	WikiPathways	Hepatitis C and Hepatocellular Carcinoma	13	9.1 × 10^−5^
hsa-miR-106b-5p	WikiPathways	Hepatitis C and Hepatocellular Carcinoma	7	2.1 × 10^−4^
hsa-miR-17-5p	WikiPathways	Hepatitis C and Hepatocellular Carcinoma	8	5.6 × 10^−4^
hsa-miR-15b-5p	WikiPathways	Hepatitis C and Hepatocellular Carcinoma	10	2.0 × 10^−3^
hsa-miR-18a-5p	WikiPathways	Hepatitis C and Hepatocellular Carcinoma	5	0.012
hsa-miR-376c-3p	WikiPathways	Hepatitis C and Hepatocellular Carcinoma	2	0.025
hsa-miR-326	WikiPathway	Hepatitis C and Hepatocellular Carcinoma	3	0.037
hsa-miR-381-3p	WikiPathways	Hepatitis C and Hepatocellular Carcinoma	30	0.049

**Table 4:** Edge betweenness of cDEG–cDEM interaction network. The type score of one indicated the gene–miRNA interaction and the type score of two indicated the gene–gene interaction. The full table is shown in Table S5.

No.	Edge	Edge Betweenness	Type
1	FOS interacts with H2AFZ	512.84	2
2	C8B interacts with FTCD	423.06	2
3	STMN1 interacts with TUBA1B	408.92	2
4	hsa-miR-1301-3p interacts with MT1F	387.96	1
5	hsa-miR-25-5p interacts with HAL	380.97	1
6	hsa-miR-331-5p interacts with FOS	377.00	1
7	hsa-miR-326 interacts with GLS2	370.50	1
8	FTCD interacts with HAL	369.99	2
9	hsa-miR-1301-3p interacts with CLEC4G	348.00	1
10	hsa-miR-25-5p interacts with STMN1	343.13	1
11	hsa-miR-326 interacts with SERPINA4	313.91	1
12	hsa-miR-2277-5p interacts with C8B	307.48	1
13	hsa-miR-1301-3p interacts with IGF1	279.78	1
14	hsa-miR-25-5p interacts with ANXA2	275.02	1
15	HAL interacts with LYVE1	270.90	2

## Data Availability

The datasets that were created during this study is publicly available in the GEO database (https://www.ncbi.nlm.nih.gov/geo) (accessed on 30 April 2022) with the accession numbers (GSE101685, GSE176271, GSE164760, GSE176288, GSE158523).

## Supplementary Materials

The following supporting information can be downloaded at https://www.mdpi.com/article/10.3390/cimb45090466/s1. Table S1: Significant DEGs in three datasets (GSE101685, GSE176271, GSE164760); Table S2: Significant DEMs in GSE176288 and GSE158523; Table S3: Full table of DEG–DEM interaction prediction; Table S4: Full table of target enrichment analysis. Table S5: Full table of edge betweenness of the interaction network.
